# Fragile X syndrome carrier screening accompanied by genetic consultation has clinical utility in populations beyond those recommended by guidelines

**DOI:** 10.1002/mgg3.1024

**Published:** 2019-11-06

**Authors:** Katherine Johansen Taber, Jeraldine Lim‐Harashima, Harris Naemi, Jim Goldberg

**Affiliations:** ^1^ Myriad Women’s Health South San Francisco CA USA; ^2^ Myriad Genetics, Inc. Salt Lake City UT USA

**Keywords:** carrier screening, fragile X syndrome, premutation

## Abstract

**Background:**

Fragile X syndrome (FXS) is the most common inherited form of intellectual disability. Many providers offer preconception or prenatal FXS carrier screening. However, guidelines recommend screening only for those with a family history or undergoing fertility evaluation. Wider screening has been resisted because of concerns about patient understanding of FXS‐associated inheritance patterns and phenotypes. Additionally, the clinical utility has been questioned.

**Methods:**

We addressed these concerns by analyzing reproductive decision‐making and pregnancy management informed by post‐test genetic consultation among 122 *FMR1* premutation carriers identified by expanded carrier screening.

**Results:**

Sixty‐three percent of those screened met guidelines screening criteria; the remaining 37% did not. Ninety‐eight percent had undergone post‐test genetic consultation. Of respondents screened preconceptionally, 74% reported planning or pursuing actions to reduce the risk of an affected pregnancy; the extent to which couples planned/pursued these actions was not significantly different between those meeting either screening criterion (76%) versus those meeting neither criterion (55%). Of respondents screened prenatally, 41% pursued prenatal diagnostic testing; the extent to which couples pursued prenatal diagnosis was not significantly different between those who met either screening criterion (37%) versus those who met neither criterion (31%).

**Conclusion:**

These results support the expansion of FXS screening criteria in guidelines.

## Graphical abstract

In this study, we report on reproductive decision‐making and pregnancy management informed by genetic consultation among a large cohort of FMR1 premutation carriers. We found that physicians recommended screening for a substantial number of patients even when they did not meet the American College of Obstetricians and Gynecologists or American College of Medical Genetics and Genomics screening criteria, and that half of patients who did not meet the screening criteria requested it. Importantly, meeting the criteria had little effect on the extent to which patients acted to reduce the risk of an affected pregnancy, calling into question the value of guidelines and coverage policies restricting screening to only those who meet the criteria

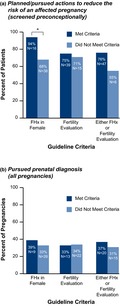

## 1 INTRODUCTION

Fragile X syndrome (FXS) is the most common inherited form of intellectual disability, caused by a region of expanded *CGG* trinucleotide repeats in *FMR1* (OMIM accession number: 309550) on the X chromosome. Males carrying more than 200 repeats (an *FMR1* “full mutation”) are almost always affected with FXS, exhibiting developmental delay and intellectual disability. Females heterozygous for a full mutation also exhibit developmental delay and intellectual disability, but with less frequency and milder severity. Males who carry a “premutation,” that is, 55–200 repeats, are not affected with FXS, but are at risk for fragile X‐associated tremor/ataxia syndrome (FXTAS), a disorder characterized by late‐onset progressive cerebellar ataxia and intention tremor. Females who are heterozygous for a premutation are also at risk for FXTAS, albeit less so than males, and are at risk for *FMR1*‐related primary ovarian insufficiency (FXPOI), resulting in reduced fertility (Saul & Tarleton, 2012).

*CGG* repeats in *FMR1* can undergo expansion in the next generation, such that females carrying a premutation are at risk for having offspring affected by FXS. The probability of expansion is dependent on the number of repeats; more than 100 repeats nearly always expands to a full mutation in the next generation (Nolin et al., 2003, 2011). The risk of expansion to a full mutation is reduced as the number of maternal repeats decreases. Very rarely in premutation carriers, the number of *CGG* repeats can contract in the next generation (Nolin et al., 2011, 2019).

Approximately 1 in 150 women carry an *FMR1* premutation that confers risk for FXS in offspring (Berkenstadt, Ries‐Levavi, Cuckle, Peleg, & Barkai, 2007; Cronister, Teicher, Rohlfs, Donnenfeld, & Hallam, 2008; Toledano‐Alhadef et al., 2001). For this reason, many providers believe that *FMR1* carrier screening should be offered to all women who are pregnant or considering pregnancy (Acharya & Ross, 2009; Archibald et al., 2013). Additionally, patients report wanting to undergo such screening (Fanos, Spangner, & Musci, 2006). However, professional society guidelines do not universally recommend screening. Though the American College of Obstetricians and Gynecologists (ACOG) states that women informed about FXS may request to undergo FXS carrier screening (ACOG, 2010), both ACOG and the American College of Medical Genetics and Genomics (ACMG) recommend offering FXS carrier screening only to those with a family history of FXS or intellectual disability suggestive of FXS, FXTAS, or a history of FXPOI (ACOG, 2010, 2017a; Sherman, Pletcher, & Driscoll, 2005). Both also recommend FXS carrier screening as part of an infertility evaluation. Many premutation carriers do not have a family history (Berkenstadt et al., 2007), leading the ACOG to acknowledge that its recommendations do not effectively identify those at risk for pregnancies affected with FXS (ACOG, 2010, 2017a).

Offering FXS carrier screening to all pregnant women and those considering pregnancy has been resisted for two main reasons. First, concerns exist about the ability to adequately counsel large numbers of screened women about the complex inheritance patterns and wide range of phenotypes associated with FXS, as well as the potential identification of those at increased risk for FXTAS or FXPOI (Sherman et al., 2005). Second, the clinical utility of FXS carrier screening has been questioned (Dimmock, 2017), focusing on the lack of an available prenatal treatment rather than the established purpose of carrier screening to inform family planning and pregnancy management (ACOG, 2017b; Edwards et al., 2015). Consequently, insurers do not cover universal FXS carrier screening, stating that it is “investigational” (Aetna, 2019; Blue Shield of California, 2019) and/or “not medically necessary” (Cigna, 2019) beyond those with a family history or who are experiencing infertility.

In this study, we sought to address the concerns about expanding FXS carrier screening to all women who are pregnant or considering pregnancy. We analyzed reproductive decision‐making and pregnancy management informed by post‐test genetic consultation among a large cohort of *FMR1* premutation carriers, some of whom met family history or fertility evaluation guideline criteria for testing and some of whom did not.

## 2 METHODS

### 2.1 Editorial Policies and Ethical Considerations

This study was reviewed and designated as exempt by the Western Institutional Review Board.

### 2.2 Study cohort

The study cohort was a subset of a larger cohort described previously (Johansen Taber et al., 2019). Briefly, the larger cohort included couples who had received carrier screening by Foresight^TM^ (Myriad Women's Health, formerly Counsyl) between 1 September 2015 and 31 December 2017, had consented to be involved in research, and were found to be at risk for current or future pregnancies affected by at least one of 176 autosomal recessive or X‐linked conditions (Hogan et al., 2018). These couples were invited to complete a survey about their actions following receipt of expanded carrier screening results. In the study reported here, invited couples were those in which the female partner was found to be an *FMR1* (NC_000023.11/Gene ID 2332) premutation carrier. Those who reported family history in the female partner as a reason for screening were considered to have a family history of FXS; however, family history was reported in a non‐condition‐specific manner, thus a participant could have had a family history for a condition other than FXS (see Discussion).

### 2.3 Survey development, fielding, and data collection

Survey questions were developed as described in Johansen Taber et al., 2019. Questions were programmed into commercial software (Logician®, Decision Analyst Inc., Arlington, TX) to optimize survey administration and response collection, and the survey was fielded between 28 February 2018 and 19 March 2018. The overall response rate for the survey was 24%. Respondents who reported being at risk for FXS in current or future pregnancies constituted the cohort reported in this study.

### 2.4 Data analysis

Descriptive statistics were used to characterize general data trends. Statistical significance between proportions was determined using chi‐square analysis; a result was considered significant when *p* < .05 at the 95% confidence level. To reduce the chance that reproductive and pregnancy management actions were a result of risk of conditions other than FXS, only those whose current or future pregnancies were at risk for FXS and no other conditions screened were included in the cohort. Patients were considered as meeting ACOG or ACMG guidelines if they reported that they underwent carrier screening as part of a fertility evaluation or because of a family history of a condition screened.

## 3 RESULTS

### 3.1 Study participants

One hundred and twenty‐two *FMR1* premutation carriers comprised the study cohort; 94% (*N* = 115) were between the ages of 20 and 40 years (Table 1). Forty percent (*N* = 49) were pregnant when they received their screening results (Table 1). Among those who were not pregnant, 47% (*N* = 34) were undergoing or planning to undergo in vitro fertilization (IVF) at the time they received their results. One hundred and three pregnancies occurred among all respondents; these pregnancies include the 49 screened prenatally and an additional 54 pregnancies subsequent to screening in both those screened preconceptionally and those screened prenatally. Respondents represented more than 11 ethnicities, with Northern European, Other/Mixed Caucasian, and Ashkenazi Jewish being the most commonly reported (Table 1). Respondents and their reproductive partners represented more than eight religions, with Jewish, Protestant, Catholic, and no affiliation most commonly reported (Table 1).

**Table 1:** Study participants

	*N* (%)
Total screened	122 (100)
Screened preconceptionally	73 (60)
Planning/pursuing IVF at time of screening	34 (47)
Screened prenatally	49 (40)
0–13 weeks pregnant	26 (53)
14–26 weeks pregnant	23 (47)
27+ weeks pregnant	0
Total pregnancies^a^	103 (100)
Age	
20–30 years	38 (31)
31–40 years	77 (63)
41–50 years	7(5.7)
Ethnicity (female)	
Northern European	44 (36)
Other/Mixed Caucasian	40 (33)
Ashkenazi Jewish	24 (20)
Southern European	11 (9.0)
Hispanic	7 (5.7)
African or African‐American	4 (3.3)
East Asian	4 (3.3)
South Asian	4 (3.3)
French Canadian or Cajun	2 (1.6)
Middle Easter	2 (1.6)
Southeast Asian	1 (0.8)
Unknown	2 (1.6)
Prefer not to say	3 (2.6)

Religion	Female *N* (%)	Male *N* (%)
Jewish	24 (20)	20 (16)
Protestant	24 (20)	15 (12)
Catholic	21 (17)	21 (17)
No affiliation	18 (15)	27 (22)
Agnostic	8 (6.6)	7 (5.7)
Atheist	5 (4.1)	8 (6.6)
Hindu	2 (1.6)	2 (1.6)
Mormon	1 (0.8)	2 (1.6)
Other	7 (5.7)	9 (7.4)
Prefer not to say	11 (9.0)	11 (9.0)

a Includes all pregnancies during which screening occurred, as well as all subsequent pregnancies in those screened prenatally and all pregnancies in those screened preconceptionally.

### 3.2 Screening delivery and consistency with guideline criteria

Seventy‐seven percent (*N* = 94) of respondents reported that their providers recommended screening, while the remaining 23% (*N* = 28) of respondents reported that they had requested screening themselves (Table 2). Provider‐recommended screening proportions were not significantly different for those who were (75%, *N* = 55) or were not (80%, *N* = 39) pregnant when screened (Table 2). Similarly, patient‐requested screening proportions were not significantly different for those who were (25%, *N* = 18) or were not (20%, *N* = 10) pregnant when screened (Table 2). Patients reported that they had undergone a post‐test genetic consultation with a genetic counselor at Myriad Women's Health (58%, *N* = 71), a local genetic counselor (50%, *N* = 61), and/or a provider other than a genetic counselor (54%, *N* = 66; Table 2; respondents could report more than one genetic consultation provider). Only two out of the 122 respondents (1.6%) reported not having undergone some form of post‐test genetic consultation (Table 2).

**Table 2:** Characteristics of screening delivery

	Total *N* (%)	Screened preconceptionally *N* (%)	Screened prenatally *N* (%)
Number screened	122 (100)	73 (100)	49 (100)
Instigation of screening			
Provider recommended	94 (77)	55 (75)	39 (80)
Met FXS screening criteria	63 (67)	51 (93)^a^	12 (31)^a^
Did not meet screening criteria	31 (33)	4 (7.3)	27 (69)
Requested by patient	28 (23)	18 (25)	10 (20)
Met FXS screening criteria	14 (50)	11 (61)	3 (30)
Did not meet screening criteria	14 (50)	7 (39)	7 (70)
Reason for screening			
Part of routine workup	37 (30)	8 (11)	29 (59)
Part of fertility workup	59 (48)	52 (71)^a^	7 (14)^a^
Female partner's ethnicity	16 (13)	9 (12)	7 (14)
Male partner's ethnicity	10 (8.2)	4 (5.5)	6 (12)
Female partner's family history	26 (21)	17 (23)	9 (18)
Male partner's family history	4 (3.3)	4 (5.5)	0
Unknown family history (either)	3 (2.5)	1 (1.4)	2 (4.1)
Post‐test genetic consultation			
Discussed with GC at MWH	71 (58)	46 (63)	25 (51)
Discussed with local GC	61 (50)	28 (38)	33 (67)
Discussed with other provider	66 (54)	43 (59)	23 (47)
None, but considering it in future	2 (1.6)	1 (1.4)	1 (2.0)
None, and not planning to in future	0	0	0

GC, genetic counselor; MWH, Myriad Women's Health.

a *p* < .05, significant difference between those screened preconceptionally and those screened prenatally.

Guidelines recommend offering screening to females with a family history of FXS or FXS‐related disorders and in those undergoing fertility evaluation (ACOG, 2010, 2017a; Sherman et al., 2005); we therefore assessed how many participants reported these as reasons for undergoing screening. Forty‐eight percent (*N* = 59) reported undergoing screening as part of a fertility evaluation; those screened preconceptionally were significantly more likely to report a fertility evaluation (71%, *N* = 52) as the reason for screening than were those screened prenatally (14%, *N* = 7; *p* < .05; Table 2). Twenty‐one percent (*N* = 26) reported undergoing screening due to the female partner's family history; those screened preconceptionally were not significantly more likely to report a family history (23%, *N* = 17) as the reason for screening than were those screened prenatally (18%, *N* = 9; *p* > .05; Table 2).

Among patients reporting that their providers had recommended screening, 67% (*N* = 63) were screened because of a family history or fertility evaluation and 33% (*N* = 31) were screened for other reasons (Table 2). Those whose providers recommended screening preconceptionally were significantly more likely to report a family history or fertility evaluation (93%, *N* = 51) than were those whose providers ordered screening prenatally (31%, *N* = 12; *p* < .05; Table 2). Among patients reporting that they had requested screening themselves, 50% (*N* = 14) reported a family history or fertility evaluation and 50% (*N* = 14) did not (Table 2). Sixty‐one percent (*N* = 11) of those who requested screening preconceptionally reported a family history or fertility evaluation, while 30% (*N* = 3) of those who requested screening prenatally reported a family history or fertility evaluation; this difference was not significantly different (*p* > .05).

### 3.3 Reproductive decision‐making and pregnancy management among *FMR1* premutation carriers

**Figure 1:**
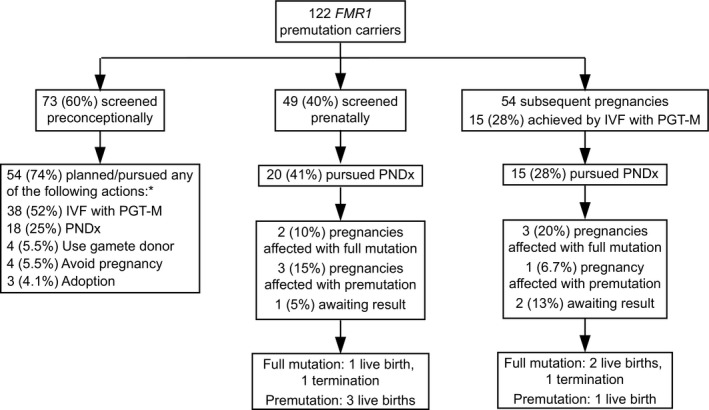
Reproductive actions and outcomes among *FMR1* (NC_000023.11/Gene ID 2332) premutation carriers. “Subsequent pregnancies” refers to pregnancies occurring subsequent to screening in both those screened preconceptionally and those screened prenatally. *Percents sum to >100% since respondents could choose more than one option. IVF: In vitro fertilization, PGT‐M: Pre‐implantation genetic testing for monogenic conditions, PNDx: Prenatal diagnosis

**Figure 2:**
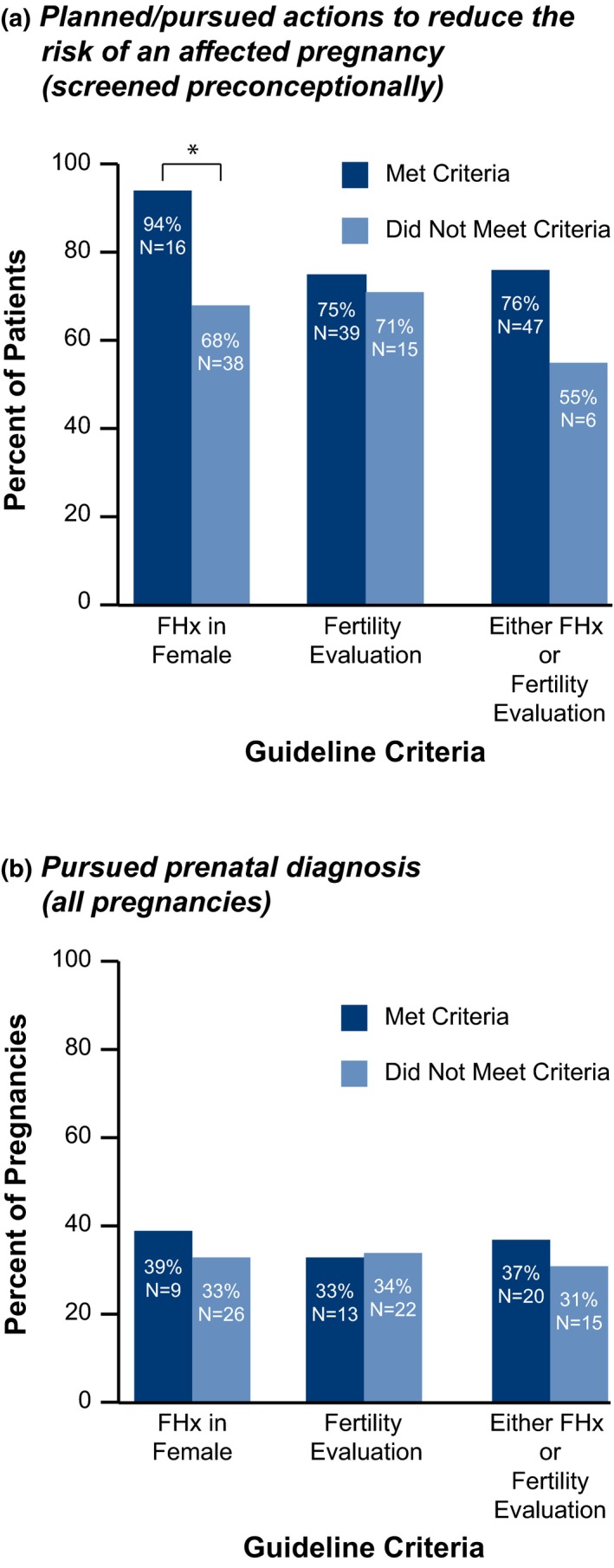
Actions taken by *FMR1 (*NC_000023.11/Gene ID 2332) premutation carriers who did or did not meet screening criteria. (a) Proportions of those screened preconceptionally who took action to reduce the risk of an affected pregnancy (including in vitro fertilization with preimplantation genetic testing for monogenic conditions, prenatal diagnostic testing once pregnant, use of a donor gamete, no longer planning to get pregnant, and adoption). (b) Proportions of pregnancies undergoing prenatal diagnostic testing. Those who met the screening criteria are indicated by dark blue; those who did not are indicated by light blue. * indicates a significant difference (*p* < .05); FHx: family history

Of the study participants screened preconceptionally (*N* = 73), 74% (*N* = 54) reported planning or pursuing actions to reduce the risk of an affected pregnancy (Figure 1). These actions included IVF with pre‐implantation genetic testing for monogenic conditions (PGT‐M; 52%, *N* = 38), prenatal diagnostic testing (PNDx; by amniocentesis or chorionic villus sampling) once pregnant (25%, *N* = 18), use of a donor gamete (5.5%, *N* = 4), no longer planning to get pregnant (5.5%, *N* = 4), and adoption (4.1%, *N* = 3; more than one action could be planned/pursued, so the overall percents equal more than 100%). Greater than two‐thirds (68%, *N* = 38) of patients who did not report a family history planned or pursued the aforementioned actions (Figure 2a); this proportion was significantly higher in those who reported a family history (94%, *N* = 16; *p* < .05; Figure 2a). The proportion that planned/pursued actions and reported fertility evaluation (75%, *N* = 39) was not significantly different than the proportion that planned/pursued actions and did not report fertility evaluation (71%, *N* = 15; *p* > .05; Figure 2a). The proportion that planned/pursued actions and reported either a family history or fertility evaluation (76%, *N* = 47) was not significantly different than the proportion that planned/pursued actions and reported neither a family history nor fertility evaluation (55%, *N* = 6; *p* > .05; Figure 2a).

Of respondents screened prenatally (*N* = 49), 41% (*N* = 20) pursued PNDx (Figure 1). In subsequent pregnancies (those occurring subsequent to screening in both those screened preconceptionally and those screened prenatally; *N* = 54), 28% (*N* = 15) pursued PNDx (Figure 1). Aggregated results of PNDx and pregnancy outcomes are noted in Figure 1. Among all pregnancies, the proportion that pursued PNDx and reported a family history (39%), fertility evaluation (33%), or either (37%) was not significantly different than the proportion that pursued PNDx and did not report a family history (33%; *p* > .05), fertility evaluation (34%; *p* > .05), or either (31%; *p* > .05; Figure 2b).

## 4 DISCUSSION

Here, we report on reproductive decision‐making and pregnancy management informed by genetic consultation among a large cohort of *FMR1* premutation carriers. We found that physicians recommended screening for a substantial number of patients even when they did not meet ACOG or ACMG screening criteria, that is, screening because of a family history of FXS or FXS‐related disorders or as part of a fertility evaluation. Further, half of the patients who did not meet these criteria requested it. Importantly, meeting the criteria had little effect on the extent to which patients acted to reduce the risk of an affected pregnancy, calling into question the value of guidelines and coverage policies restricting screening to only those who meet the criteria.

The widely accepted purpose of carrier screening is to inform family planning and pregnancy management according to patients’ individual values (ACOG, 2017b; Edwards et al., 2015). The clinical utility of FXS carrier screening can therefore be measured by its impact on reproductive decision‐making and management of at‐risk pregnancies. Our study demonstrates clinical utility: approximately three‐quarters of those screened preconceptionally took action to reduce the risk of an affected pregnancy, and nearly half of those screened prenatally pursued PNDx. These findings are consistent with previous studies illustrating the clinical utility of FXS carrier screening. In a study of 22 pregnant women identified by prenatal carrier screening to be at high risk of pregnancies affected with FXS, 16 (72%) chose to undergo PNDx (Archibald et al., 2018). In another study, 59% of parents of children diagnosed with FXS reported that the diagnosis changed their plans to have additional children, with most deciding not to conceive (Bailey, Skinner, & Sparkman, 2003). Our study further demonstrates that the clinical utility of FXS carrier screening is not restricted to only those who reported a family history or fertility evaluation. Preconceptionally, more than two‐thirds of at‐risk patients who did not meet the family history criterion, and nearly three‐quarters who did not meet the fertility evaluation criterion, took action to reduce the risk of an affected pregnancy. Prenatally, at‐risk couples who did not meet screening criteria were as likely to pursue PNDx as were those who did meet the criteria. These findings indicate that even at‐risk couples who do not meet the screening criteria make impactful reproductive and pregnancy management decisions based on results and support the expansion of criteria to include all women who are pregnant or considering pregnancy.

Guidelines have raised concern that universally offering FXS carrier screening will result in the need to counsel large numbers of patients about the complex inheritance patterns and range of phenotypes of FXS and *FMR1* premutation carriers during an era in which the genetic counseling workforce may be experiencing a shortage (Hoskovec et al., 2018; Sherman et al., 2005). However, in our study nearly all patients underwent genetic consultation, some from more than one provider type, suggesting that they did not encounter barriers to accessing genetic counseling. Several studies have reported on mechanisms for providing genetic counseling that can accommodate more patients than can traditional in‐person counseling (Arjunan et al., 2019; Burgess, Carmany, & Trepanier, 2016; McCuaig et al., 2018). The most common are web‐based and telegenetic counseling, both of which appear to be as effective at providing posttest education as is in‐person genetic counseling (Biesecker et al., 2018; Schwartz et al., 2014). In our study, more than half of the participants utilized post‐test telephone consultations with board‐certified genetic counselors provided by the testing laboratory. A growing body of evidence demonstrating that non‐traditional counseling mechanisms are an effective alternative suggests that concern over the inability to accommodate counseling for large numbers of patients as a result of universal FXS carrier screening is less valid than it may once have been.

Calls for population‐wide carrier screening have persisted for many years as studies have revealed the shortcomings of family history‐based criteria (Metcalfe, Delatycki, Cohen, Archibald, & Emery, 2018; Pesso et al., 2000; Toledano‐Alhadef et al., 2001). Our study showed that a substantial proportion of premutation carriers did not have a family history of FXS (79%). Similarly, 80% of FXS premutation carriers studied in Archibald et al. did not have a family history (Archibald et al., 2018). Berkenstadt et al. reported that the *FMR1* premutation carrier frequency in those with a family history of intellectual disability, developmental delay, or autism (1 in 150) was not significantly different than in those without a family history (1 in 158; Berkenstadt et al., 2007). Rajendra et al. found that following ACOG family history screening guidelines would have identified fewer than half of the FXS carriers in its study (Rajendra, Bringman, Ward, & Phillips, 2008). Studies such as these are the basis for the ACOG acknowledgment that its current recommendations are insufficient for detecting all premutation carriers (ACOG, 2010, 2017a).

Other concerns with population‐wide FXS carrier screening have centered around the inability to provide precise risk estimates due to the uncertainty of *CGG* repeat expansion, and, as a result, imprecise phenotypic predictions for affected individuals. Studies on large numbers of premutation carriers have enabled more accurate prediction of repeat expansion and resulting phenotype (Berkenstadt et al., 2007; Kraan et al., 2018; Nolin et al., 2003, 2011). In addition, the recent availability of *CGG* interruption testing has allowed for refined risk estimates (Ardui et al., 2018; Latham, Coppinger, Hadd, & Nolin, 2014; Yrigollen et al., 2012). The concern that the inheritance pattern of FXS is complicated and likely difficult for some patients to understand is valid (Finucane et al., 2012; Musci & Moyer, 2010), but it is noteworthy that 98% of patients in our study underwent genetic consultation, suggesting that their reproductive and pregnancy management decisions were informed by health care professionals who could explain the inheritance pattern and provide risk estimates. Increased access to genetic counseling through the alternative mechanisms discussed above may address the need for patient education.

This study has limitations to consider. It relied on patient recall of actions resulting from carrier screening; patient memory can sometimes be inaccurate. In addition, those who planned or pursued actions based on FXS carrier screening results may have been more willing than those who did not to report on such actions via the survey. Conversely, some premutation carriers may have declined to complete the survey out of reluctance to share difficult pregnancy management decisions. For those reporting that they underwent carrier screening as part of a fertility evaluation, we cannot rule out that carrier screening was ordered as part of routine care for those planning pregnancy rather than as part of the infertility assessment. However, because the survey question asked patients *why* they underwent screening, we considered it to be the latter. As no difference was seen in the extent of action undertaken by those who did or did not report a fertility evaluation as the reason for screening, we believe that parsing respondents based on more stringent fertility evaluation criteria would not change the conclusion. Finally, the survey reported in this study was designed to capture actions resulting from carrier screening for a number of conditions (Johansen Taber et al., 2019). Thus, when a respondent reported family history as the reason for undergoing carrier screening, it is possible that she may have had a family history of conditions other than FXS. However, this would have resulted in an overestimate of those reporting family history for FXS; the exact proportion would likely have been lower than the 21% of female premutation carriers reporting family history in this cohort, strengthening our conclusion that many patients who do not meet family history criteria used their results to make meaningful reproductive and pregnancy management decisions. A study with patients carrying an *FMR1* premutation, and with a confirmed positive or negative family history of FXS, FXTAS, or FXPOI, could further explore the similarity in the extent of actions undertaken between the two groups.

## 5 CONCLUSION

We demonstrate here that providers recommend, and patients request, FXS carrier screening outside of guidelines criteria, and that patients take action to reduce the risk of an affected pregnancy regardless of whether they meet the criteria for screening. Further, patients’ actions were informed by genetic consultation. Our study adds support to the expansion of FXS carrier screening to include all women who are pregnant or considering pregnancy.

## CONFLICT OF INTEREST

All authors are current employees of Myriad Women's Health or Myriad Genetics, Inc.

## AUTHOR CONTRIBUTIONS

Katherine Johansen Taber, Harris Naemi, and Jim Goldberg all made substantial contributions to the conception, design, and interpretation of data. All authors made a substantial contribution to the acquisition and analysis of data. Katherine Johansen Taber, Jeraldine Lim‐Harashima, and Jim Goldberg were involved in the drafting and/or editing of the manuscript. All authors gave final approval of the manuscript and agree to be accountable for all aspects of the work.

## DATA AVAILABILITY STATEMENT

The data that support the findings of this study are available from the corresponding author upon reasonable request.
